# Differential expression patterns of purinergic ectoenzymes and the antioxidative role of IL-6 in hospitalized COVID-19 patient recovery

**DOI:** 10.3389/fimmu.2023.1227873

**Published:** 2023-09-25

**Authors:** Yanina Luciana Mazzocco, Gastón Bergero, Sebastian Del Rosso, Natalia Eberhardt, Claudia Sola, Héctor Alex Saka, Sofía María Villada, José Luis Bocco, Maria Pilar Aoki

**Affiliations:** ^1^ Consejo Nacional de Investigaciones Científicas y Técnicas (CONICET), Centro de Investigaciones en Bioquímica Clínica e Inmunología (CIBICI), Córdoba, Argentina; ^2^ Universidad Nacional de Córdoba, Facultad de Ciencias Químicas, Departamento de Bioquímica Clínica, Córdoba, Argentina; ^3^ Servicio de Enfermedades Infecciosas, Hospital Privado Universitario de Córdoba, Córdoba, Argentina

**Keywords:** CD39, CD73, nitric oxide, cytokines, inflammatory monocytes, SARS-CoV-2, nitrotyrosine

## Abstract

**Introduction:**

We have acquired significant knowledge regarding the pathogenesis of severe acute respiratory syndrome caused by coronavirus 2 (SARS-CoV-2). However, the underlying mechanisms responsible for disease recovery still need to be fully understood.

**Methods:**

To gain insights into critical immune markers involved in COVID-19 etiopathogenesis, we studied the evolution of the immune profile of peripheral blood samples from patients who had recovered from COVID-19 and compared them to subjects with severe acute respiratory illness but negative for SARS-CoV-2 detection (controls). In addition, linear and clustered correlations between different parameters were determined.

**Results:**

The data obtained revealed a significant reduction in the frequency of inflammatory monocytes (CD14+CD16+) at hospital discharge vs. admission. Remarkably, nitric oxide (NO) production by the monocyte compartment was significantly reduced at discharge. Furthermore, interleukin (IL)-6 plasma levels were negatively correlated with the frequency of NO+CD14+CD16+ monocytes at hospital admission. However, at the time of hospital release, circulating IL-6 directly correlated with the NO production rate by monocytes. In line with these observations, we found that concomitant with NO diminution, the level of nitrotyrosine (NT) on CD8 T-cells significantly diminished at the time of hospital release. Considering that purinergic signaling constitutes another regulatory system, we analyzed the kinetics of CD39 and CD73 ectoenzyme expression in CD8 T-cells. We found that the frequency of CD39+CD8+ T-cells significantly diminished while the percentage of CD73+ cells increased at hospital discharge. *In vitro*, IL-6 stimulation of PBMCs from COVID-19 patients diminished the NT levels on CD8 T-cells. A clear differential expression pattern of CD39 and CD73 was observed in the NT+ vs. NT-CD8+ T-cell populations.

**Discussion:**

The results suggest that early after infection, IL-6 controls the production of NO, which regulates the levels of NT on CD8 T-cells modifying their effector functions. Intriguingly, in this cytotoxic cell population, the expression of purinergic ectoenzymes is tightly associated with the presence of nitrated surface molecules. Overall, the data obtained contribute to a better understanding of pathogenic mechanisms associated with COVID-19 outcomes.

## 1 Introduction

Since coronavirus disease 2019 (COVID-19) was reported in Wuhan, China, the international scientific community has achieved remarkable advances regarding the pathogenesis of severe acute respiratory syndrome caused by coronavirus 2 (SARS-CoV-2). However, the complete map of etiopathogenic mechanisms that determine disease recovery has not been fully depicted.

The pathophysiology of severe SARS-CoV-2 infection is conducted by aggressive inflammatory responses implicated in the resulting damage to the airways (1). Therefore, disease severity is related to both the viral infection and the host response. Indeed, SARS-CoV-2 induces a dysregulated systemic hyperinflammatory response in severe-to-critical patients characterized by the abnormal secretion of inflammatory cytokines whose main marker is interleukin (IL)-6 (2). As a logical consequence, IL-6 signaling inhibition was postulated as a potential tool for reducing the inflammatory burden of COVID-19, as earlier studies observed an improvement in the clinical status of patients (3, 4). However, several randomized controlled trials of the anti-IL-6 receptor (IL-6R) antibody tocilizumab in patients with COVID-19 have shown conflicting results (5–8) and the routine use of tocilizumab in patients with moderate to severe COVID-19 is not generally supported (9). In this regard, the consequences of IL-6 blockade in COVID-19 may vary depending on the infection stage and the host’s immune status (10). Considering that in several experimental *in vivo* models of viral lung infections, IL-6 exhibits either pathogenic (11) or protective (12) effects, the role of this cytokine in SARS-CoV-2 infection should be carefully evaluated.

IL-6 was discovered in the middle of the 80s (13). Cloning of the IL-6 cDNA revealed that numerous laboratories have studied this cytokine, which is associated with different, sometimes divergent, biological activities (14). Indeed, IL-6 is a pleiotropic cytokine that modulates the immune response at different levels and mediates several distinct pathophysiological processes depending on the activated signaling pathways and cell types (15). Early after infection, IL-6 conducts the synthesis of acute phase proteins (including C-reactive protein (CRP), fibrinogen, serum amyloid protein, and haptoglobin, among others) in the liver, which orchestrate the systemic host immune response independently of the site where infection takes place (16). This notion highlights the relevant role of IL-6 at the beginning of the immune response.

Our group has exhaustively studied the role of IL-6 in the setting of a chronic infectious disease (17–19). We have reported that IL-6 released early after systemic infection with *Trypanosoma (T.) cruzi* parasite exerts a potent antioxidative effect that regulates the lethal release of nitric oxide (NO) by monocytes (18). The infection triggers the activation of macrophage NADPH oxidase, resulting in a continuous production of superoxide anion ${\text{O}}_{2}^{-}$ that stimulates infected macrophages to produce NO. NO is crucial for the defense against intracellular pathogens, but it must be tightly controlled to avoid excessive detrimental oxidative stress. In this sense, we have reported that IL-6 regulates inflammasome activation and, consequently, IL-1β-induced NO production in a murine model of *T. cruzi* infection. The anti-inflammatory action of IL-6 seems to be central for controlling cardiac and systemic oxidative stress, promoting cellular rescue from apoptosis, and protecting infected IL-6-knockout mice against death (18). The mechanism demonstrates that the protective effect of IL-6 against oxidative stress plays a crucial role in regulating the pro-oxidative environment in the early stages of infection.

The presence of NO rapidly initiates a reaction with superoxide anion-producing peroxynitrites (20). In turn, peroxynitrites cause nitration and nitrosylation of tyrosines from components of the T cell receptor (TCR) signaling complex, among other proteins in the plasma membrane, thereby controlling T-cell activation and effector functions (21, 22). The presence of nitrotyrosine (NT) and nitrotyrosine-modified proteins has been reported in several inflammatory diseases and is widely used as a hallmark of *in situ* inflammation (23). Nitration of proteins on CD8 T-cells is involved in myeloid-derived suppressor cells (MDSC)-induced CD8 T-cell tolerance (24) and a decrease in CD8 T-cell cytotoxic effector functions (19). We have demonstrated that monocyte-derived NO causes protein nitration of CD8 T-cells limiting their cytotoxic functions (19). Although a significant body of evidence highlights the role of protein nitration on the surface of CD8 T-cells (25, 26), the relationship between NO production by circulating monocytes and NT on CD8 T-cells in COVID-19 patients has not been evaluated before.

The purinergic signaling system represents an additional regulatory network for CD8 T-cells. Under inflammatory conditions, adenosine triphosphate (ATP) is released by stressed or damaged cells to the extracellular milieu (eATP) and provides proinflammatory signals to immune cells. Ectonucleoside triphosphate diphosphohydrolase 1 (ENTPD1, or CD39) catalyzes the phosphohydrolysis of eATP and generates adenosine monophosphate (AMP), which is in turn used by the ecto-5’-nucleotidase CD73 to synthesize adenosine. Thus, CD39 and CD73 ectonucleotidases play a dominant role in maintaining the balance between proinflammatory ATP and adenosine diphosphate (ADP) nucleotides and immunosuppressive adenosine. Although different reports highlight the importance of purinergic signaling in driving COVID-19 outcome (27–29) there are no data analyzing the evolution of purinergic ectoenzyme expression in cytotoxic T-cells in hospitalized recovered patients.

In the present study, we aimed to investigate the kinetics of relevant circulating immune markers modified at hospital admission and discharge in COVID-19 patients, which were comparatively analyzed with subjects diagnosed with similar symptoms of pneumonia but negative for SARS-CoV-2 detection (controls). Moreover, we determined the correlations between plasma IL-6 levels, the frequency of NO-producing monocyte subpopulations, and the rate of protein nitration of CD8 T-cells. We also explored the impact of IL-6 on T-cell tyrosine nitration and the expression of purinergic ectoenzymes in cultured peripheral blood mononuclear cells (PBMCs) from COVID-19 patients. The data obtained not only contribute to a better understanding of the etiopathogenic mechanisms associated with COVID-19 outcomes but also strengthen the significance of IL-6’s biological role in the acute response to infection.

## 2 Materials and methods

### 2.1 Subjects and ethics statement

A total of 66 patients diagnosed with COVID-19 were recruited between September 2020 and September 2021 at the Hospital Privado Universitario de Córdoba, Córdoba (Argentina). For the present prospective study, 27 patients (Group I) were selected since they had been admitted and discharged from the hospital with complete clinical data and all biological samples collected for further study. In addition, 11 hospitalized patients (Group II) diagnosed with similar clinical symptoms but negative for SARS-CoV-2 detection were used as controls. The samples and clinical data were obtained at admission and discharge (**Table 1**). The study was approved by the Comité Institucional de Ética de la Investigación en Salud del Adulto (CIEIS), Ministerio de Salud de la Provincia de Córdoba (HP 4-329). All studies were conducted according to the principles expressed in the Declaration of Helsinki. Signed informed consent documents were obtained from each donor enrolled in the study.

**Table 1:** Clinical and laboratory findings of the study cohort.

	COVID-19			Control			Pairwise Comparisons
	Women	Men	Total	Women	Men	Total	-
**N**	9 (25%)	18 (75%)	27 (100%)	5 (46.5%)	6 (54.5%)	11 (100.0%)	–
Age Category [n (%)]							
30-49	4 (44.4%)	4 (22.2%)	8 (29.6%)	1 (20.0%)	1 (16.7%)	2 (18.2%)	–
50-70	3 (33.3%)	9 (50.0%)	12 (44.4%)	2 (40.0%)	3 (50.0%)	5 (45.5%)	–
>70	2 (22.2%)	5 (27.2%)	7 (26.0%)	2 (40.0%)	2 (33.3%)	4 (36.3%)	–
Comorbidities							
n (%)	7 (77.8%)	16 (88.8%)	23 (85.1%)	4 (80.0%)	4 (66.7%)	8 (72.2%)	*P = 0.415 †P = 0.816 ‡P = 0.324
median (IQR)	3 (3)	2 (2)	2 (2)	2 (2)	1 (2)	2 (2)	
**Hb (g%)**	12.7 ± 0.83	14.39 ± 2.05	13.50 ± 1.75	10.27 ± 1.68	11.61 ± 4.31	11.08 ± 3.43	*P = 0.024 †P = 0.053 ‡P = 0.256 ^&^P = 0,089 ^#^P = 0,156
**CRP (mg·L^-1^)**	6.77 ± 4.35	8.26 ± 5.67	7.70 ± 5.17	14.17 ± 6.81	16.64 ± 15.11	15.65 ± 12.00	*P = 0.071 †P = 0.482 ‡P = 0.770 ^&^P = 0,036 ^#^P = 0,241
**SatO_2_ (%)**	89.4 ± 4.0	85.5 ± 11.4	87.44 ± 8.9	93.2 ± 2.2	93.3 ± 2.2	93.3 ± 2.1	*P = 0.003 †P = 0.359 ‡P = 0.955 ^&^P = 0,116 ^#^P = 0,027
Pneumonia Severity [n (%)]							
Mild	–	1 (5.6%)	1 (3.7%)	–	–	–	–
Moderate	9 (100%)	16 (88.9%)	25 (92.6%)	4 (80.0%)	6 (100.0%)	10 (90.9%)	–
Severe	–	1 (5.6%)	1 (3.7%)	1 (20.0%)	–	1 (9.1%)	–
**Days of Hospitalization**	8,6 ± 3,7	7,6 ± 4,7	8 ± 4,3	*NA*	*NA*	*NA*	†P = 0.594
**ICU**	2 (22.2%)	6 (33.3%)	8 (29.6%)	*1 (20%)*	*0 (0%)*	*1 (9.1%)*	†P = 0.016

COVID-19 individuals with a positive SARS Cov-2 diagnosis and control individuals diagnosed with non-COVID-19 severe acute respiratory illness. Data are expressed as absolute and relative frequency, mean ± SD or median and interquartile range (IQR). Hb, hemoglobin; CRP, C-reactive protein; SatO_2_, oxygen saturation; ICU, Intensive care unit; NA, not available *Comparisons between COVID-19 vs. Control, †Comparisons between women vs. men within COVID-19 group, ‡Comparison between women vs. men within the Control group, ^&^Comparisons between women in COVID-19 group vs. Control group, and ^#^Comparisons between men in COVID-19 group vs. Control group.

Criteria used for recruitment: Group I: hospitalized adult patients (> 18 years old) who qualified as a confirmed case of COVID-19 (a case with laboratory confirmation of infection by RT-qPCR). Group II: adult patients (> 18 years old) who were suspected cases of COVID-19 with negative laboratory test (RT-qPCR). A suspected case of COVID-19 (30) was defined as a patient with severe acute respiratory illness (measured fever >38°C, and at least one sign/symptom of respiratory disease, e.g., cough, shortness of breath; AND requiring hospitalization) in the absence of an alternative diagnosis that fully explains the clinical presentation.

### 2.2 Blood samples

Approximately 10 mL of blood were drawn from each patient by venipuncture and placed into heparinized tubes. PBMCs were isolated through density gradient centrifugation using Ficoll^®^-Paque™ PLUS (GE Healthcare Bioscience) and frozen in freezing media that consisted of heat-inactivated fetal bovine serum (Natocor) and 10% DMSO (Gibco). Both plasma and cells were stored in clearly labeled aliquots at -80°C. All blood samples were collected between 6 and 8 am. Plasma and cells from all peripheral blood samples were processed within 5h of the blood samples being taken.

### 2.3 Quantification of cytokines

Plasma samples were analyzed for IL-1β, IL-6, tumor necrosis factor (TNF)-α, IP-10, IL-8 (CXCL-8), GM-CSF, IL-12p70, interferon (IFN)-α, IFN-β, IFN-γ, IFN-λ1, IFN-λ2/3, and IL-10 levels by using a bead-based multiplex assay (#740349, LEGENDplex™ Biolegend) and flow cytometry (FACS Canto II, BD Biosciences), according to the manufacturer’s instructions. Data were analyzed by LEGENDplex™ software after performing the standard curves.

### 2.4 Cell subpopulation analysis

PBMCs were thawed and washed twice with PBS. Then, the cells were stained with the following anti-human antibodies: PE-Cy7-CD3, PE/Dazzle594-CD4, APC-Cy7-CD8a, PE-Cy7-CD14, PerCP-Cy5.5-CD16, BV421-CD39, PE-CD73 (BioLegend), anti-nitrotyrosine (anti-NT) rabbit (Cat. #BS-8551R, Bioss Thermo Fisher) and Zombie Aqua (BioLegend). Nitric oxide production was evaluated using the molecular probe DAF-FM DA (10 μM, Cat. #D23844 Invitrogen). The oxidized product was measured at excitation/emission wavelengths of 488/520 nm. Labeled samples were acquired using a BD LSRFortessa FACS cytometer, and data were analyzed using FlowJo™ v10 software (Tree Star, Inc.). The compensation matrixes were designed using UltraComp eBeads™ Compensation Beads (01-222-42; Invitrogen) with specific markers.

### 2.5 *Ex vivo* IL-6 stimulation

PBMCs from COVID-19 patients obtained at admission were thawed and washed twice with PBS. Then, the cells were cultured in supplemented RPMI with recombinant human bioactive IL-6 (20 pg/mL or 20 ng/mL) (Cat. #570802, BioLegend) or maintained in medium alone. After 48 h, live cells were selected by Zombie Aqua exclusion, and CD4+ cells, CD8+ cells, and CD14+ monocytes were analyzed for NT staining, CD39/CD73 expression, or NO production by flow cytometry.

### 2.6 Statistical analysis

Data are expressed as mean and standard deviation or median and interquartile range according to their distribution. The normality of distributions was assessed employing the Shapiro-Wilk test and visual inspection of Q-Q plots and box plots. Outliers were identified using studentized residuals and visual inspection of box plots. A value was considered an outlier if it was ≥ 3 or ≤ -3 studentized units. Regarding the outlier exclusion criteria employed, if an outlier did not significantly affect the results or the assumptions behind the statistical test it was not removed. Those variables showing a non-normal distribution were log-transformed (natural logarithm) to normalize their distribution. Variables with a non-normal distribution despite transformation were analyzed using non-parametric methods (Mann-Whitney U Test and Kruskal-Wallis). Pairwise comparisons between COVID-19 patients and controls were performed using t-tests for independent samples, while pairwise comparisons between patients’ admission and release were performed using paired-sample t-tests. Hospitalization days were modeled using univariate general linear models adjusting for potential covariates such as sex, age, and comorbidities. A model was considered valid if the following assumptions were met: (i) independence of observations (i.e., independence of residuals); (ii) a linear regression between the dependent variable and each of the independent variables and between the dependent variable and the independent variables collectively; (iii) homoscedasticity of residuals; (iv) multicollinearity; (v) significance of outliers, and (vi) normal distribution of errors. If one or more of these assumptions was violated, then several corrections were applied in an attempt to solve the problem. The percentage of variance explained by each model was assessed using the adjusted determination coefficient (r^2^) and the Akaike and Bayesian Information Criteria (AIC and BIC) and Hosmer-Lemshow goodness of fit test were used. The homogeneity of variance was assessed using the Levene test. In the case of a significant difference in variance homogeneity, the Brown-Forsythe robust test of equality of means was used with Dunnett’s T3 *post hoc* pairwise comparisons. Bivariate correlations between variables were computed using Pearson’s or Spearman’s coefficients. Principal component analysis (PCA) with a Varimax rotated matrix and Kaiser normalization was performed to evaluate whether variables such as cytokines, NT+ CD8+ T-cells, or NO-producing monocytes could discriminate between COVID-19 patients at admission, at discharge, and controls. The analyses were performed using SPSS software for Windows^®^ (Version 20.0; Armonk, USA) or R with the Performance Analytics and Factoextra Package, while figures were drawn employing Prisma GraphPad v9.0 (GraphPad, San Diego, CA, USA). All analyses were conducted with an alpha level of 0.05. Statistical power was computed *a posteriori* and reported when appropriate.

## 3 Results

### 3.1 Participants

The twenty-seven COVID-19 patients included nine women (57.3 ± 13.8 yrs.) and eighteen men (59.1 ± 9.2 yrs.) with no significant differences in hospitalization days, number of comorbidities, hemoglobin (Hb), CRP or oxygen saturation (SatO_2_) between the sexes (**Table 1**). Twenty-three patients had at least one comorbidity with a range from one to six. Women exhibited a higher concentration of IFN-γ at admission (17.95 ± 13.68 vs. 6.35 ± 7.52 pg·mL^-1^, P = 0.013) with no other differences in inflammatory markers between sexes. In addition, no significant differences were observed between women and men regarding the frequencies of lymphocyte and monocyte populations or in NT+ lymphocytes and NO+ monocytes. Of the twenty-seven COVID-19 patients, eight spent varied time in the intensive care unit (ICU) (from 4 to 25 days), and two of them had severe evolution of the disease. Only one from the control group was admitted in ICU.

The following comorbidities were registered in 1).-COVID-19 patients: 13/27 metabolic disorders (diabetes, overweight-obesity, dyslipidemia), 5/27 cancers (pancreatic, colon, anal, breast, acute myeloid leukemia), 10/27 pulmonary disorders (asthma, pulmonary fibrosis, Chronic Obstructive Pulmonary Disease [COPD], smoking), 17/27 cardiovascular conditions (arrhythmia, ischemic cardiomyopathy, acute myocardial infarction, arterial hypertension), and 9 others (gastritis, hypothyroidism, psoriasis, renal transplant, osteoarthritis, psychiatric disorders); and 2).-Control group: 1/11 metabolic disorder (diabetes), 2/11 cancers (breast, biliary tract), 4/11 pulmonary disorders (asthma, smoking), 5/11 cardiovascular conditions (ischemic cardiomyopathy, arterial hypertension, heart failure), and 4/11 others (hypothyroidism, renal transplant, Chronic Kidney Disease [CKD], ulcerative colitis).

### 3.2 Kinetics of plasma levels of immune markers found in COVID-19 patients at hospital admission and discharge

**Figure 1:**
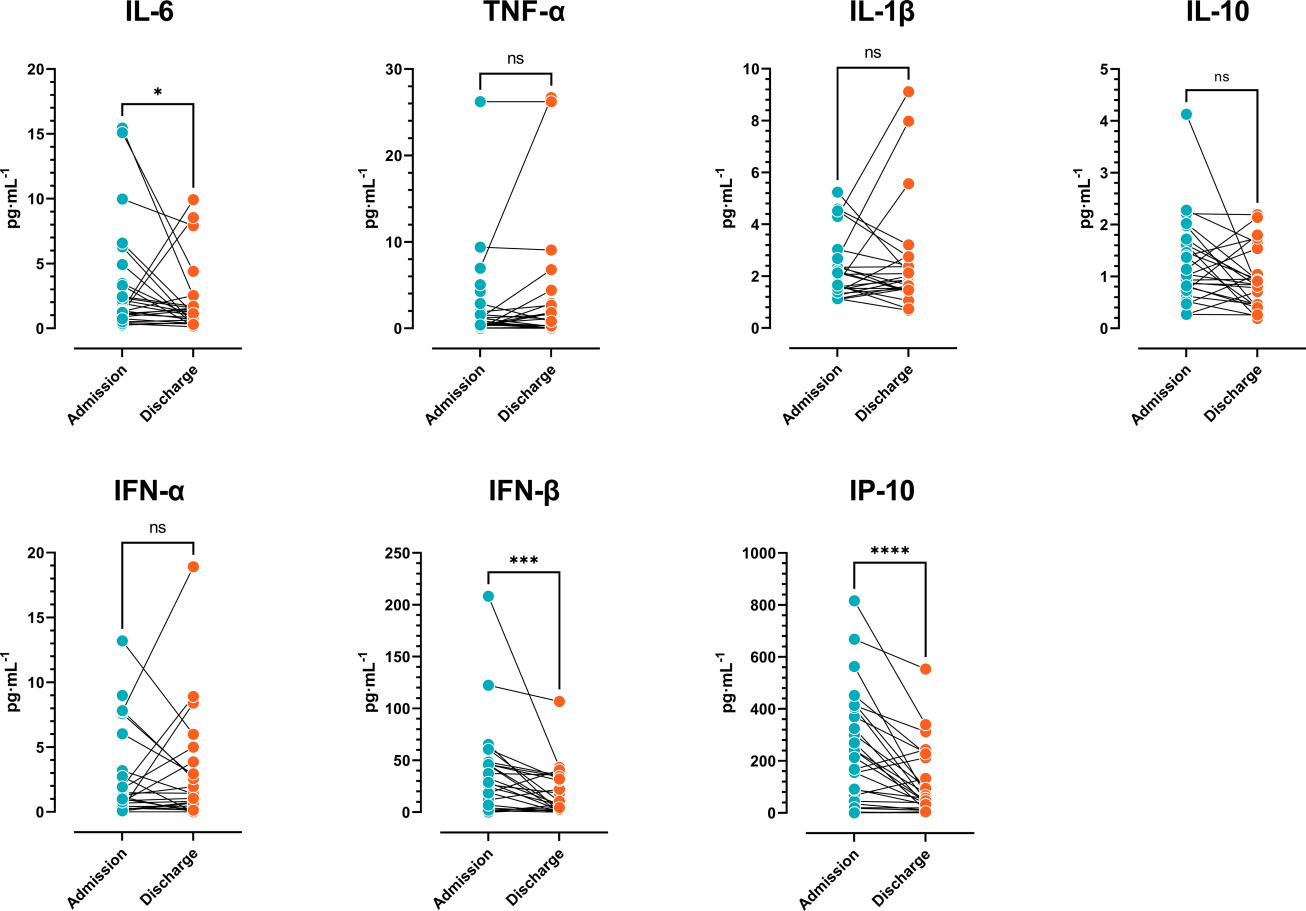
Cytokine plasma levels. Comparison of cytokine concentrations of COVID-19 patients at admission vs. discharge (n=27) (Wilcoxon signed-rank test). The results are expressed as mean ± SD (ns: not significant; * p < 0.05; ** p < 0.01; *** p < 0.001).

As shown in **Figure 1**, there was a significant reduction in IL-6, IFN-β, and IP-10 plasma levels in COVID-19 patients at hospital admission vs. discharge. These data indicated that following an early increase in cytokines, COVID-19 patients with moderate disease displayed a progressive reduction in the type-1 (antiviral) response.

### 3.3 Kinetics of monocyte populations in COVID-19 patients at hospital admission vs. discharge

**Figure 2:**
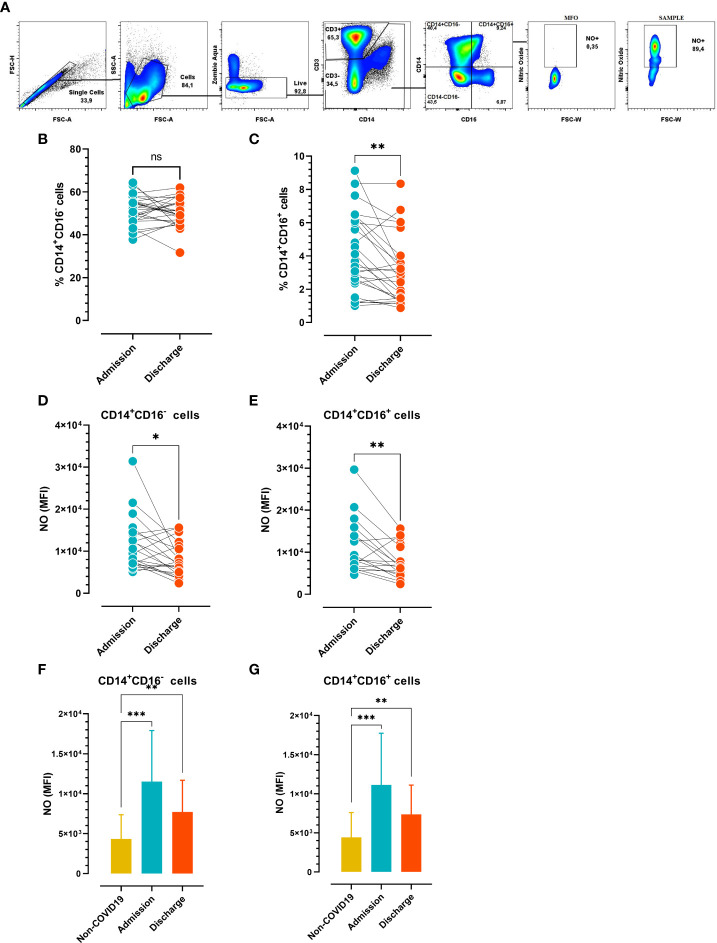
Frequency of circulating monocyte profiles at admission vs. discharge **(A)** Gate strategy analysis employed to determinate nitric oxide (NO) produced by monocytes. **(B)** Frequency of circulating CD14+CD16- and **(C)** CD14+CD16+ cells (n=26). **(D, E)** Mean Fluorescence Intensity **(**MFI) of NO-producing monocyte subsets (n=21). **(B–E)** Paired comparisons (admission vs. discharge) were analyzed using the Wilcoxon test. **(F, G)** MFI of NO-producing monocyte subsets in COVID-19 patients at hospital admission and discharge (n=21) vs. non-COVID-19 subjects (n=10). Unpaired comparisons (i.e., admission vs. non-COVID-19 or release vs. non-COVID-19) were analyzed using the Mann-Whitney test. The results are expressed as mean ± SD (* p < 0.05; ** p < 0.01; *** p < 0.001).

Human peripheral blood monocytes can be divided into a major CD14+CD16- population and a minor CD14+CD16+ subpopulation. The number of circulating CD16+ monocytes drastically increases in inflammatory conditions with respect to regular monocytes (CD14+CD16−) (19). To study the monocyte compartment in the setting of SARS-CoV-2 infection, we examined the frequency of monocyte subpopulations and NO-producing monocyte subsets at hospital admission vs. discharge in COVID-19 patients. Following the gating strategy shown in **Figure 2A**, the monocytes were classified as non-inflammatory (CD14+CD16-) or inflammatory (CD14+CD16+) subsets. There was a significant reduction in the percentages of CD14+CD16+ at admission vs. discharge, and no significant difference in the frequency of CD14+CD16- cells (**Figures 2B, C**). Interestingly, the Mean Fluorescence Intensity (MFI) of NO was significantly different at admission vs. discharge for the two monocyte subsets (**Figures 2D, E**). Furthermore, there were significant differences in NO production in both monocyte subpopulations at admission and release vs. the same populations in control donors (**Figures 2F, G**). Of note, the small number of subjects in the control group (N=11) would have limited the statistical analysis. These findings indicated that circulating monocytes increase the production of NO early after infection independent of the subset analyzed, but this production is substantially reduced at the time of hospital discharge.

**Figure 3:**
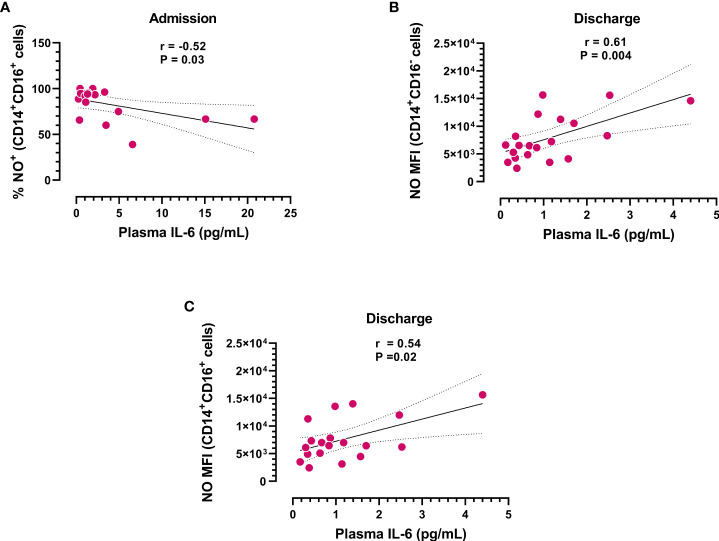
Correlation analysis between IL-6 plasma levels and subpopulations of NO+ monocytes. **(A)** Frequency of CD14+CD16+ monocytes producing NO at admission (n=18) and **(B, C)** NO production in different monocyte subsets at hospital discharge (n=20) *vs.* IL-6 plasma concentrations. Dotted lines represent the confidence interval for the correlation coefficient.

Considering the antioxidative role of IL-6, we next analyzed the possible correlation between IL-6 plasma levels and NO produced by different monocyte subsets. We found that IL-6 plasma levels inversely correlated with the frequency of NO+CD14+CD16+ monocytes at hospital admission (**Figure 3A**). In contrast, circulating IL-6 levels directly correlated with the rate of NO production in the monocyte subsets studied at hospital discharge (**Figures 3B, C**), suggesting that the levels of IL-6 could influence the effector function of monocytes.

### 3.4 Analysis of cytokine levels and circulating cell populations vs. the length of hospital stay

When the length of hospital stays and cytokine levels were modeled using univariate general linear models adjusted for potential confounders (age, sex, and comorbidities), significant adjusted models were obtained for IL-6, TNF-α, IP-10, IL-8, and IL-10 (**Table 2**). In addition, the analysis of cytokine level variations (Δ) between studied times, also showed significant models for IP-10, IL-10, and IFN-γ. The results suggest that the plasma levels of inflammatory (IL-6, TNF-α, IP-10 and IL-8), and anti-inflammatory cytokines (IL-10 and IL-6) at admission predict the length of hospitalization. Moreover, a significant adjusted model was obtained between hospitalization days and the frequency of NO+CD14+CD16+ cells [B = -0.075, SE = 0.033, 95% CI (-0.146 to -0.004), η^2^ = 0.25, 1-β = 0.56].

**Table 2:** Univariate general linear models for hospitalization days and cytokine plasma levels adjusted for potential confounders (age, sex, and comorbidities).

Cytokines	Regression Slope	SE	95% Confidence Interval		η^2^	1-β
			Lower	Upper		
**IL-6**	0.081	0.020	0.040	0.123	0.42	0.97
**TNF-α**	0.211	0.047	0.113	0.308	0.48	0.99
**IP-10**	0.006	0.002	0.003	0.010	0.37	0.94
**IL-8**	0.181	0.048	0.082	0.280	0.38	0.95
**IL-10**	0.709	0.145	0.410	1.008	0.51	0.99
**ΔIP-10**	-0.006	0.001	-0.009	-0.004	0.52	0.99
**ΔIL-10**	-0.635	0.139	-0.923	-0.346	0.49	0.99
**ΔIFN-γ**	0.108	0.045	0.013	0.203	0.24	0.61

SE, standard error; η^2^ = effect size, 1-β = statistical power.

### 3.5 Analysis of the association between NT+CD8+ T-cells and the production of NO by monocytes

**Figure 4:**
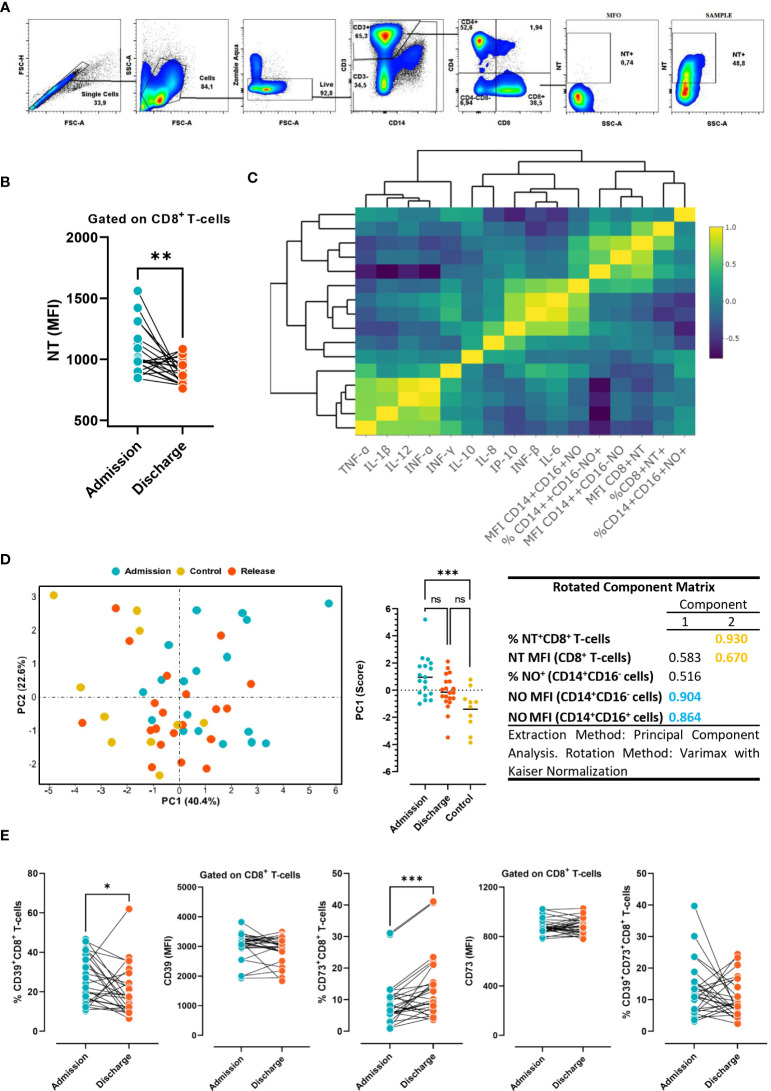
Levels of NT and purinergic ectoenzymes in CD8 T cells. **(A)** Gate strategy employed to determine the MFI of nitrotyrosine (NT) in CD8 T-cells from COVID-19 patients. **(B)** Levels of NT in circulating CD8 T-cells in COVID-19 patients at admission and discharge (n=26). Wilcoxon test. **(C)** Clustered correlation matrix between NO production by monocytes, NT+CD8+ T-cell, and cytokine plasma concentration at hospital admission. **(D)** Principal component analysis (PCA), based on NO production in monocytes and NT in CD8 T-cells in COVID-19 patients at hospital admission and discharge, and non-COVID-19 patients. **(E)** Frequency and expression levels of CD39 and CD73 on CD8 T- cells at hospital admission *vs*. discharge (n=26). The results are expressed as mean ± SD (* p < 0.05; ** p < 0.01; *** p < 0.001).

We next sought to test the hypothesis that NO production by monocytes could increase the level of protein nitration of CD8 T-cells, and this would differ between the studied conditions (admission, release, and controls with non-COVID-19 severe acute respiratory illness). Following the gating strategy depicted in **Figure 4A**, it was observed that the MFI of nitrotyrosine (NT) on CD8 T-cells was significantly reduced at discharge compared to admission in COVID-19 subjects (**Figure 4B**). Furthermore, the clustered correlation analysis showed a positive correlation between the percentage of NO-producing monocytes and the production of NO by monocyte subsets and the MFI of nitrated CD8 T-cells (**Figure 4C**). In line with this, principal component analysis (PCA) was able to discriminate between patients at admission and those in the control group (**Figure 4D**), with the MFI of NO+CD14+CD16- cells and the MFI of NO+CD14+CD16+ cells as the most weighted variables in PC1, and the frequency of NT+CD8+ T-cells and the MFI of NT+CD8+ cells in PC2. The results clearly suggest that NO production by monocytes could influence the nitration level of CD8 T-cells at hospital admission.

Considering that the purinergic system is a potent immune regulatory system, we also sought to comparatively determine the expression levels of CD39 and CD73 ectoenzymes in CD8 T-cells at hospital admission and discharge. We found that while the frequency of cytotoxic cells expressing CD39 significantly diminished, CD73 expression significantly increased at hospital discharge in comparison with hospital admission (**Figure 4E**). The results suggest that immune cells activate different purinergic signaling pathways at the time of admission and discharge. The above observations support the hypothesis that CD39 upregulation on conventional T cells prevents uncontrolled inflammation by limiting the availability of eATP released in response to acute infection. In turn, during the resolution of the infection, CD73 increases to warrant the anti-inflammatory action of adenosine.

### 3.6 Differential expression patterns of CD39 and CD73 on NT+CD8+ T-cells in *in vitro* cultured PBMCs from COVID-19 patients

**Figure 5:**
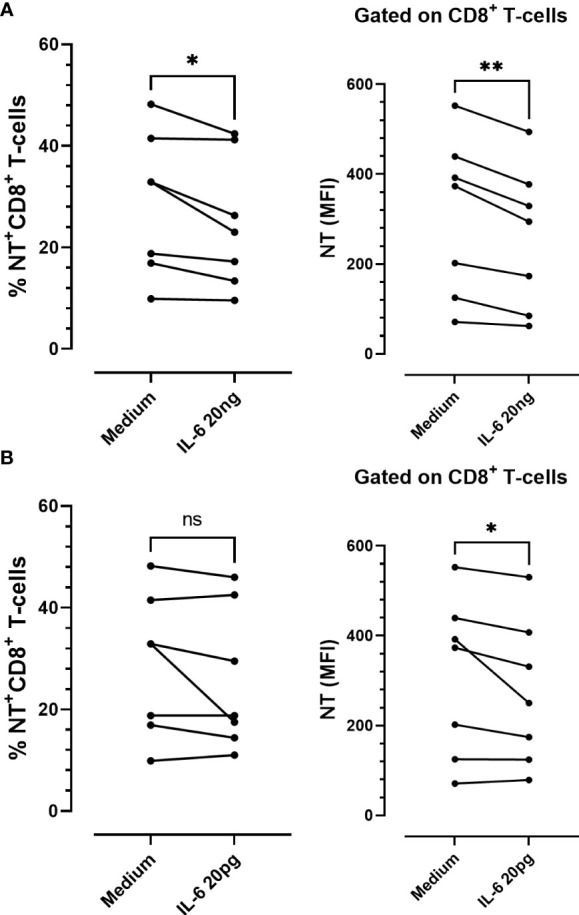
*In vitro* stimulation of PBMCs from COVID-19 patients with recombinant IL-6. Frequency and expression levels of nitrotyrosine (NT) in CD8+ T-cells stimulated with **(A)** 20 ng/mL or **(B)** 20 pg/mL of IL-6 for 48 h (n = 7) (ns: not significant; * p < 0.05; ** p < 0.01; *** p < 0.001).

**Figure 6:**
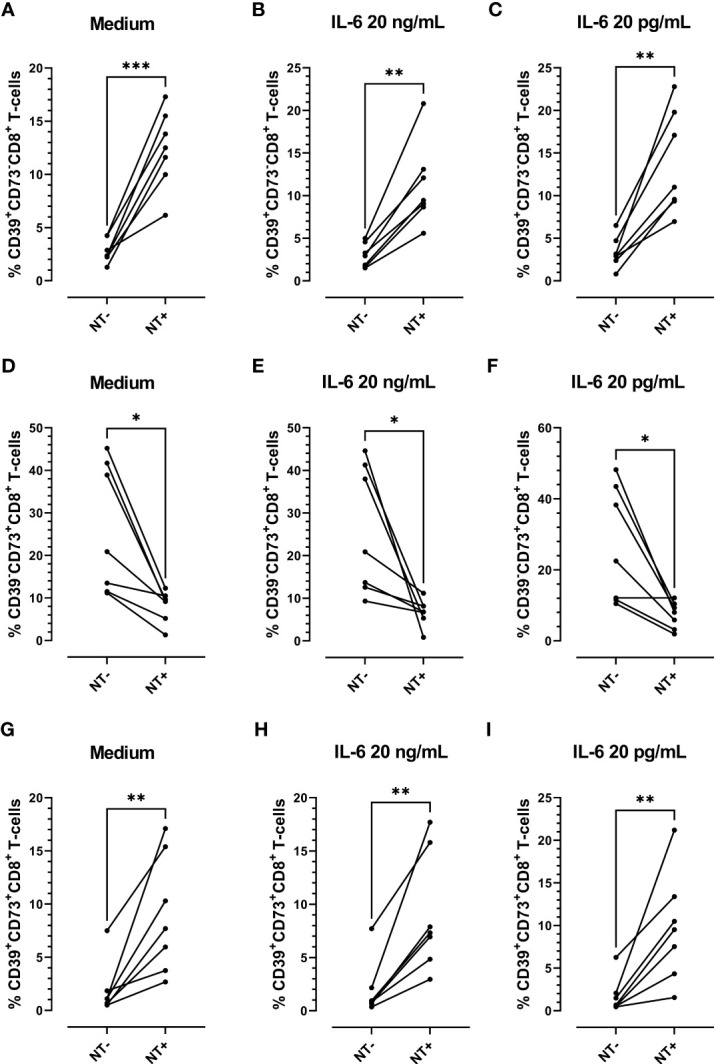
Analysis of the expression of purinergic ectoenzymes in CD8 T-cells stimulated *in vitro* with recombinant IL-6. Frequency of CD39+CD73-CD8+ T-cells **(A)** unstimulated (medium) or stimulated with **(B)** 20 ng/mL or **(C)** 20 pg/mL of IL-6. Frequency of CD39-CD73+ CD8+ T-cells **(D)** unstimulated (medium) or stimulated with **(E)** 20 ng/mL or **(F)** 20 pg/mL of IL-6. Frequency of CD39+CD73+CD8+ T-cells **(G)** unstimulated (medium) or stimulated with **(H)** 20 ng/mL and **(I)** 20 pg/mL of IL-6 for 48 h (n = 7) (* p < 0.05; ** p < 0.01; *** p < 0.001).

Considering the antioxidative effect of IL-6 we aimed to establish the impact of this cytokine on tyrosine nitration and CD39 and CD73 expression patterns, using *in-vitro*-cultured PBMCs obtained from COVID-19 patients. PBMCs were stimulated with IL-6 or maintained in medium alone for 48 h. Two different IL-6 concentrations were employed: a) 20 pg/mL (similar to the maximum plasma level detected in the total (66) COVID-19 patients recruited; Media= 22,88 pg/mL), and b) 20 ng/mL (the plasmatic concentration in severe-to-critical COVID-19 disease, recommended for tocilizumab treatment (31)). We found that IL-6 stimulation significantly diminished the levels of NT on CD8 T-cells, irrespective of the concentration assayed. However, at the higher concentration (20 ng/mL), IL-6 also significantly diminished the frequency of NT+CD8+ T-cells (**Figures 5A, B**), suggesting that higher levels of IL-6 are required to decrease the percentage of NT+ cells. Remarkably, the frequency of NT+CD8+ T-cell expressing CD39 was significantly higher than the frequency of CD39+ cells in the NT- counterpart, while the opposite was found for CD73+ cells (**Figure 6**). Furthermore, the frequency of double-positive (CD39+CD73+) cells was higher in NT+CD8+ T-cell and NT+CD4+ T-cell analyzed populations than in their NT- counterparts (**Figures 6G–I**; **Supplementary Figure 1**). These observations were independent of IL-6 stimulation since the same effect was observed when the cultures were subjected to the IL-6 treatments or maintained in medium alone. Of note, IL-6 did not modify the frequency of CD39+ and CD73+ cells when total CD8 and CD4 T-lymphocyte populations were analyzed (**Supplementary Figure 2**). The results clearly evidence that nitrotyrosilation of cytotoxic cells is associated with the expression pattern of purinergic ectoenzymes.

## 4 Discussion

Our analysis of the immune profile of peripheral blood samples from COVID-19 patients at hospital admission and discharge reveals temporal features of the immune response to SARS-CoV-2 infection and helps to better understand the biology of disease recovery and to identify potential predictors.

Regarding soluble mediators, when comparing cytokine plasma levels in COVID-19 patients at hospital admission vs. discharge, there was a significant reduction in cytokines associated with the antiviral response, but the levels of Th1 cytokines and the anti-inflammatory cytokine IL-10 did not exhibit significant differences. The results suggest that hospitalized COVID-19 patients display a progressive reduction in the antiviral response, but Th1 and anti-inflammatory cytokines are delayed in reaching basal levels. Of note, plasma levels of some inflammatory, but also anti-inflammatory cytokines at the time of admission allowed the prediction of hospitalization length, highlighting the importance of cytokine networks in defining disease outcomes.

Considering that dysregulated cytokine release is a major contributing factor to the development of severe COVID-19, acting on the cytokine cascade is a frequently implemented strategy for preventing disease progression in hospitalized patients. Among anti-cytokine therapies, pharmacological blockers of the IL-6 signaling pathway have been the most commonly used interventions, with different degrees of success. The COVINTOC study, a randomized, controlled, phase 3 trial performed in India, did not support the routine use of anti-IL-6R antibody (tocilizumab) in adults with moderate to severe COVID-19 (9). More recently, based on the analysis of observational studies, randomized controlled trials, and meta-analyses, Zizzo and colleagues proposed a window for therapeutic interventions with IL-6 inhibitors. They found that therapy with anti-IL-6R monoclonal antibodies (e.g., tocilizumab, sarilumab) seems to be effective in severe disease and in a particular window of time (31). Antiviral therapies may be helpful in the early stages of the disease to control virus replication. However, immunomodulatory and anti-cytokine therapies aimed at targeting hyperinflammatory reactions should be established later to prevent severe deterioration and allow the cytokine network’s beneficial effects. Therefore, a deep understanding of the biology of cytokine responses is essential to select the right drug and timing for effective treatment (32, 33). Early after infection, IL-6 drives hepatic synthesis and secretion of acute-phase reactants, which orchestrate the systemic immune response. Remarkably, IL-6 exerts negative feedback mechanisms on proinflammatory cytokines by suppressing their production (e.g., TNF) (34), increasing the number of their decoy receptors (e.g., sTNFRp55, IL-1RA) (35) or inducing negative intracellular regulators (36, 37), and inhibiting natural killer and cytotoxic CD8 T-cell release of perforin and granzymes (38). Thus, in the early stages of COVID-19, in which IL-6 plays protective roles, IL-6R inhibition would not be helpful but would be detrimental. Indeed, tocilizumab was observed to provoke a transitory increase in IFN-γ and a substantial increase in IL-6 and IL-10 levels (39). In the present study, we found that IL-6 plasma levels were significantly reduced in recovered patients compared to those at hospital admission. Given these results and considering the antioxidative effect of IL-6 previously reported by our group and others, we proposed an additional regulatory mechanism through which IL-6 may play essential roles during the early stages of SARS-CoV-2 infection.

Monocytes are essential in fighting viral pathogens, and SARS-CoV-2 infection is no exception (40). In humans, monocytes are classified based on CD14 and CD16 expression. CD14+CD16- monocytes, are the most common subtype and can potentially become CD103+ dendritic cells in the lung. Their elevated levels of the migratory receptor CCR2 facilitate their exit from the bone marrow and rapid movement to sites of inflammation or infection (41). On the other hand, the inflammatory CD14+CD16+ population can develop into CD11b+ dendritic cells and can become macrophages in the lung (42, 43). Reports of alterations in the number and kinetics of circulating monocyte subsets during coronavirus infection depend on the stage of the disease and are variable in different research articles (40, 44). In the present study, we found a significant reduction in the frequency of inflammatory monocytes (CD16+) at discharge vs. admission, while the percentage of CD16- monocytes did not undergo modifications. Remarkably, NO production by the monocyte compartment was significantly reduced at discharge, independent of the subset analyzed. Furthermore, NO production was increased at admission and release compared with each subset in control donors. The data suggest that independent of the subset, circulating monocytes increase NO production early after infection, but it is controlled at the time of hospital discharge.

With respect to monocytes and COVID-19 outcome, Zhou and colleagues investigated the pathological role of CD14+CD16+ monocytes in patients with severe pulmonary syndrome (45). They demonstrated that during coronavirus infection, CD4 T-cells shift to pathogenic Th1 cells, which produce GM-CSF and secrete a large amount of IL-6. In turn, CD14+CD16+ monocytes require the presence of GM-CSF to initiate tissue damage in both mice and humans (46, 47). Therefore, the entrance of pathogenic GM-CSF+ Th1 cells and inflammatory CD14+CD16+ monocytes into the lung circulation is considered a clue step in inducing pulmonary immunopathology and acute mortality after SARS-CoV-2 infection. In agreement with this notion, a significantly higher frequency of CD14+CD16+ monocytes was found in the peripheral blood of COVID-19 patients compared to healthy donors. The percentage was much higher in severe pulmonary-syndrome patients. Remarkably, significantly higher production of IL-6 was detected in these monocytes, making the inflammatory storm even worse (45). Based on these results, targeting GM-CSF or IL-6 with a monoclonal antibody has been proposed as a potential treatment strategy to reduce the harmful immune response caused by SARS-CoV-2 infection (48). In addition, other research teams identified the presence of the CD14+CD16+ monocyte subset in blood samples of individuals with COVID-19, which exhibited a significant capability to contribute to cytokine storms by producing substantial amounts of TNF-α, IL-10, and IL-6 that are linked to the deterioration of patients (49, 50). Understanding the role of CD14+CD16+ monocytes in COVID-19 pathogenesis is crucial for developing effective therapies to manage the cytokine storm and prevent severe disease outcomes. Remarkably, in addition to the increased frequency of NO-producing monocytes, we found that the plasma levels of IL-6 inversely correlated with the frequency of NO+CD16+CD14+ monocytes at hospital admission of infected patients. In agreement with this observation, we have reported that IL-6 mediates the regulation of NO production induced in response to a systemic infection (18). Indeed, IL-6 has a critical role in regulating inflammasome activation and IL-1β-induced NO production early after pathogen encounter in a murine model of *T. cruzi* infection (the causal agent of Chagas disease). We found that excessive oxidative stress accounts for the increased mortality observed in infected IL-6-deficient mice (51). The IL-6 antioxidant response was also observed in *in vitro*-infected peripheral blood mononuclear cells obtained from healthy donors (18) and other human pathologies (52). In contrast to the common belief that IL-6 is primarily a proinflammatory component, an important body of evidence assigns IL-6 crucial roles in regulating the immune response and protecting against oxidative stress (53–57). It has been suggested that this apparent discrepancy in IL-6 action may result from cell type–specific effects, differences in acute versus chronic processes, or the inflammatory microenvironment, among other factors. In the present work, we observed that at the time of hospital release, the plasma levels of IL-6 directly correlated with the rate of NO production by the monocyte subsets. The data suggest that early after infection, high levels of IL-6 exert an antioxidative effect on inflammatory monocytes. In contrast, IL-6 loses this property at lower levels, suggesting that the effect also depends on the amount of circulating cytokine and likely on the composition of the inflammatory milieu.

The NO produced by monocytes reacts with the superoxide anion-generating peroxynitrites that induce the nitration of surface proteins on CD8 T-cells, an effect involved in a decrease in CD8-cytotoxic effector functions (19). In line with this notion, the level of NT on CD8 T-cells significantly diminished at the time of hospital discharge, concomitant with the reduction in NO-producing monocytes. After principal component analysis and cluster correlation studies, we conclude that NO produced by monocytes may influence the NT level of CD8 T-cells in COVID-19 patients. Thus, the decrease in the oxidative capacity of circulating monocytes at the time of hospital discharge could influence the diminution of tyrosine nitration on the surface of CD8 T-cells. In this sense, the recovery of patients could be attributed to the restoration of CD8 cytotoxic activity. It is widely accepted that in inflammatory conditions, MDSCs play a significant role in regulating immune responses, maintaining immune balance, and preventing overactivation of the immune system. Through the production of peroxynitrite and reactive oxygen species (ROS), MDSCs induce CD8 T-cell tolerance by disrupting peptide–MHC–TCR binding through the nitration of the TCR–CD8 complex (24). In COVID-19 patients, there is an early expansion of circulating MDSCs (58). In addition, MDSCs frequency correlated with the level of inflammatory mediators and their frequency was persistently higher in patients with severe disease than in those with mild disease (59). In agreement with the decrease in surface nitration of CD8 T-cells observed in our study, the authors reported that the frequency of MDSCs declined at the convalescent phase in patients with mild disease. Although further studies are required to define the role of CD8 T-cell tyrosine nitration, its diminution could be considered a biomarker of disease recovery.

Purinergic signaling plays a significant role in infectious diseases and is involved in regulating immune responses and modulating inflammation (60). Recently it was reported that patients with severe COVID-19 exhibited higher levels of CD39+ T-cells, while the frequency of CD4 and CD8 T-cells expressing CD73 was decreased (29). Similarly, another group demonstrated that severe COVID-19 patients exhibited higher frequencies of CD39+CD4+ T-cells and diminished frequencies of CD73+CD4+ and CD73+CD8+ T-cells in comparison with mild COVID-19 patients and healthy controls (28). The higher expression of CD39 together with lower CD73 on T-cells may explain the diminished extracellular levels of both ATP and adenosine and could contribute to the exacerbated inflammatory state in severe COVID-19 patients, as adenosine plays a critical role in regulating inflammation (28). Remarkably, Wang and colleagues (27) identified increased CD39 expression in the lung, liver, spleen, and PBMCs from severe COVID‐19 patients. The levels of CD39 correlated with the number of days the patients spent in the hospital, days in the intensive care unit, and markers of blood coagulation. The data suggest that there may be an association between CD39 and disease progression. In line with this notion, we found that the frequency of CD8 T-cells expressing CD39 diminished significantly while the percentage of CD73+ cells increased at hospital discharge. Although further studies are required to clarify the role of CD39 and CD73 expression kinetics in T-cell populations, it seems that CD39 downregulation concomitant with CD73 upregulation contributes to COVID-19 patient recovery. Consistently, it was reported that human CD8 T-cells, following *in vitro* anti-CD3 monoclonal antibody stimulation, acquired surface expression of CD39 (61). In contrast, CD73 expression was high under basal conditions but dropped to background levels at day 5 post-stimulation. Coincidently, in a murine model of infection, naive CD8 T-cells expressed only marginal levels of CD39, but five days post-infection, the frequency of CD39+CD8+ T-cells, as well as the mean CD39 expression level, increased. Regarding CD73 expression, the majority of CD8 T-cells were positive in the uninfected mouse, but following infection, the MFI and frequency of positive cells, decreased (62). These results expand previous results obtained with human T cells (63, 64). Thus, it is possible that in COVID-19 subjects circulating CD8 T-cells exhibit the activated profile of purinergic ectoenzymes at the time of hospitalization (CD39^high^ and CD73^low^) but this pattern returns to the basal or homeostatic levels (CD39^low^ and CD73^high^) in recovered patients. Collectively, the data substantiate the possibility that in response to acute infection, CD39 is upregulated on conventional T cells to prevent uncontrolled inflammation by promoting eATP metabolization. Notably, during the resolution phase, CD73 increases to ensure the anti-inflammatory effects of adenosine.

In addition to TCR engagement, IL-6 and IL-27 exposure further promote CD39 expression in human CD8 T-cells. Moreover, recombinant IL-6 significantly induced CD39 expression on the human NK cell surface, while the IL-6-receptor antagonist tocilizumab prevented this effect (65) Collectively, the studies and findings presented indicate that blocking IL-6 in SARS-CoV-2 therapy could potentially have negative effects. This is because IL-6 blockade may interfere with the beneficial eATP metabolism accomplished by CD39 upregulation, which plays a crucial role in managing the immune response during infection.

To further evaluate the impact of IL-6 on tyrosine nitration and pattern expression of purinergic ectoenzymes, we performed *in-vitro* experiments in which cultured PBMCs from COVID-19 patients were stimulated with two IL-6 concentrations. In agreement with our previous report, which demonstrated the antioxidative role of IL-6 in cultured PBMCs (66), we found that the levels of NT on CD8 T-cell significantly diminished upon IL-6 stimulation. Remarkably, the frequency of NT+CD8+ T-cell expressing CD39 was significantly higher than that of their NT- counterparts, while the opposite was found for CD73+ cells. The results suggest that the induction of NT on CD8 T-cells is associated with CD39 expression and a diminution in CD73 expression. Our group plans to focus on conducting additional studies to understand the biological implications of these observations.

Summing up, our findings provide compelling evidence for the dynamic interplay of critical immune components during COVID-19 recovery, emphasizing the potential role of IL-6 and purinergic signaling regulatory effects that could influence the inhibition of disease progression and concomitant patient recovery.

## Data availability statement

The raw data supporting the conclusions of this article will be made available by the authors, without undue reservation.

## Ethics statement

The studies involving humans were approved by Comité Institucional de Ética de la Investigación en Salud del Adulto (CIEIS), Ministerio de Salud de la Provincia de Córdoba (HP 4-329). The studies were conducted in accordance with the local legislation and institutional requirements. The participants provided their written informed consent to participate in this study.

## Author contributions

YM, GB, and NE contributed to the study design and performed the experiments. YM, GB, and SD organized the database, performed the statistical analysis, and wrote sections of the manuscript. SV performed the recruitment and clinical evaluation of the patients. NE revised the manuscript. HS, CS, and JB conceived the project and collaborated with the general development of the study. MA conceived the project, designed, provided overall direction for the study, and wrote the first draft of the manuscript. All authors contributed to the article and approved the submitted version.
