# Supplementary material for: Differential expression patterns of purinergic ectoenzymes and the antioxidative role of IL-6 in hospitalized COVID-19 patient recovery
Source: Front Immunol. 2023 Sep 25;14:1227873. doi: 10.3389/fimmu.2023.1227873 (PMC10560791; doi:10.3389/fimmu.2023.1227873)
Supplement: Supplementary file 1 [file Presentation_1.pdf]

Differential expression patterns of purinergic ectoenzymes and the antioxidative role of IL-6 in hospitalized COVID-19 patient recovery

Yanina Luciana Mazzocco[1,2], Gastón Bergero[1,2], Sebastian Del Rosso[1,2], Natalia Eberhardt[1,2], Claudia Sola[1,2], Héctor Alex Saka[1,2], Sofía María Villada[3], José Luis Bocco[1,2] Maria Pilar Aoki[1,2]

[1]Consejo Nacional de Investigaciones Científicas y Técnicas (CONICET), Centro de Investigaciones en Bioquímica Clínica e Inmunología (CIBICI), Córdoba, Argentina
[2]Universidad Nacional de Córdoba, Facultad de Ciencias Químicas, Departamento de Bioquímica Clínica, Córdoba, Argentina
[3]Servicio de Enfermedades Infecciosas, Hospital Privado Universitario de Córdoba, Córdoba, Argentina

Supplementary Figures

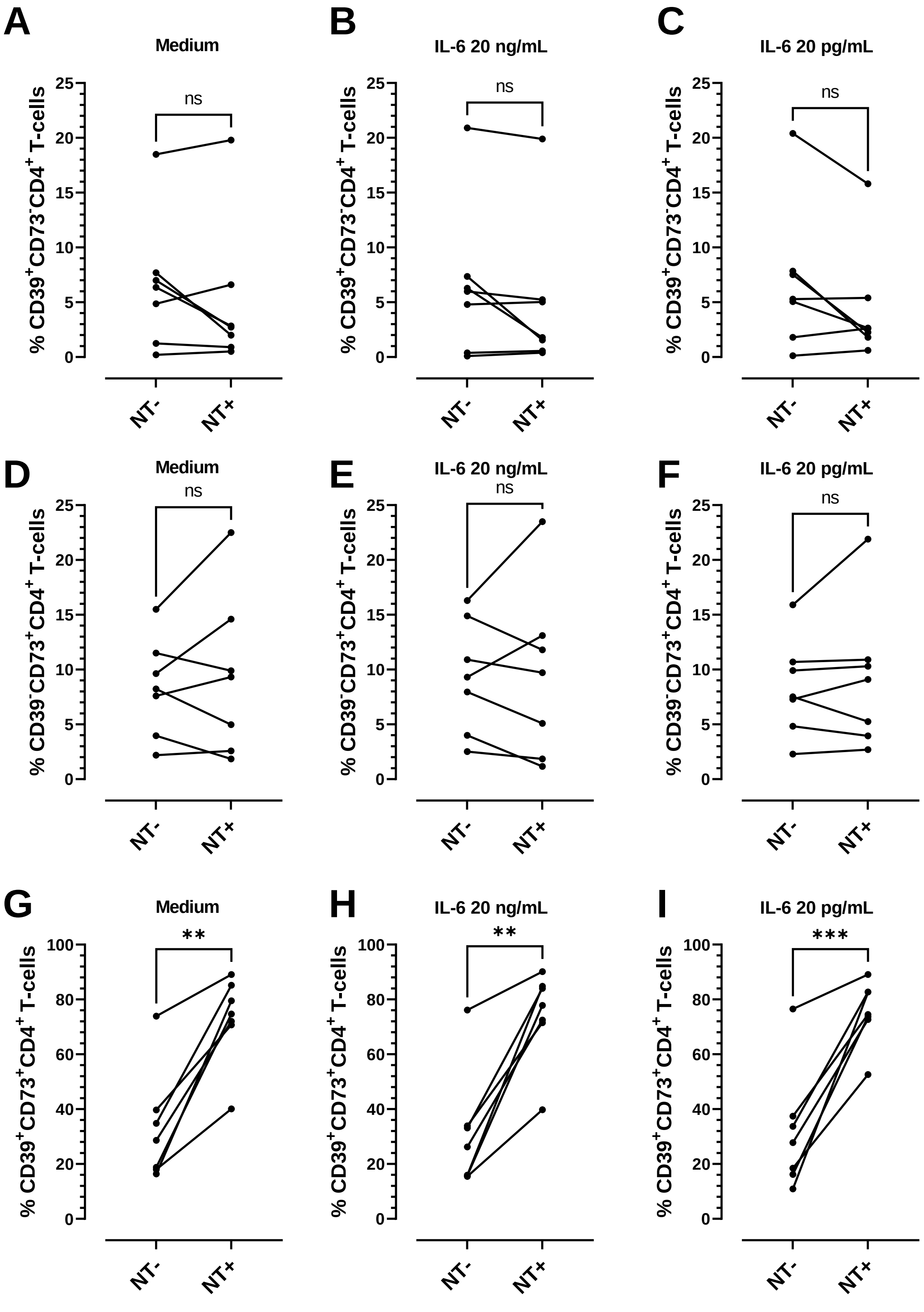

Supplementary Figure 1. Frequency and expression levels of CD39 and CD73 in NT+ or NT- CD4+ T-cells stimulated with recombinant IL-6 (20 ng/mL or 20 pg/mL) or maintained in medium. ns: not significant; * p < 0.05; ** p < 0.01; *** p < 0.001.

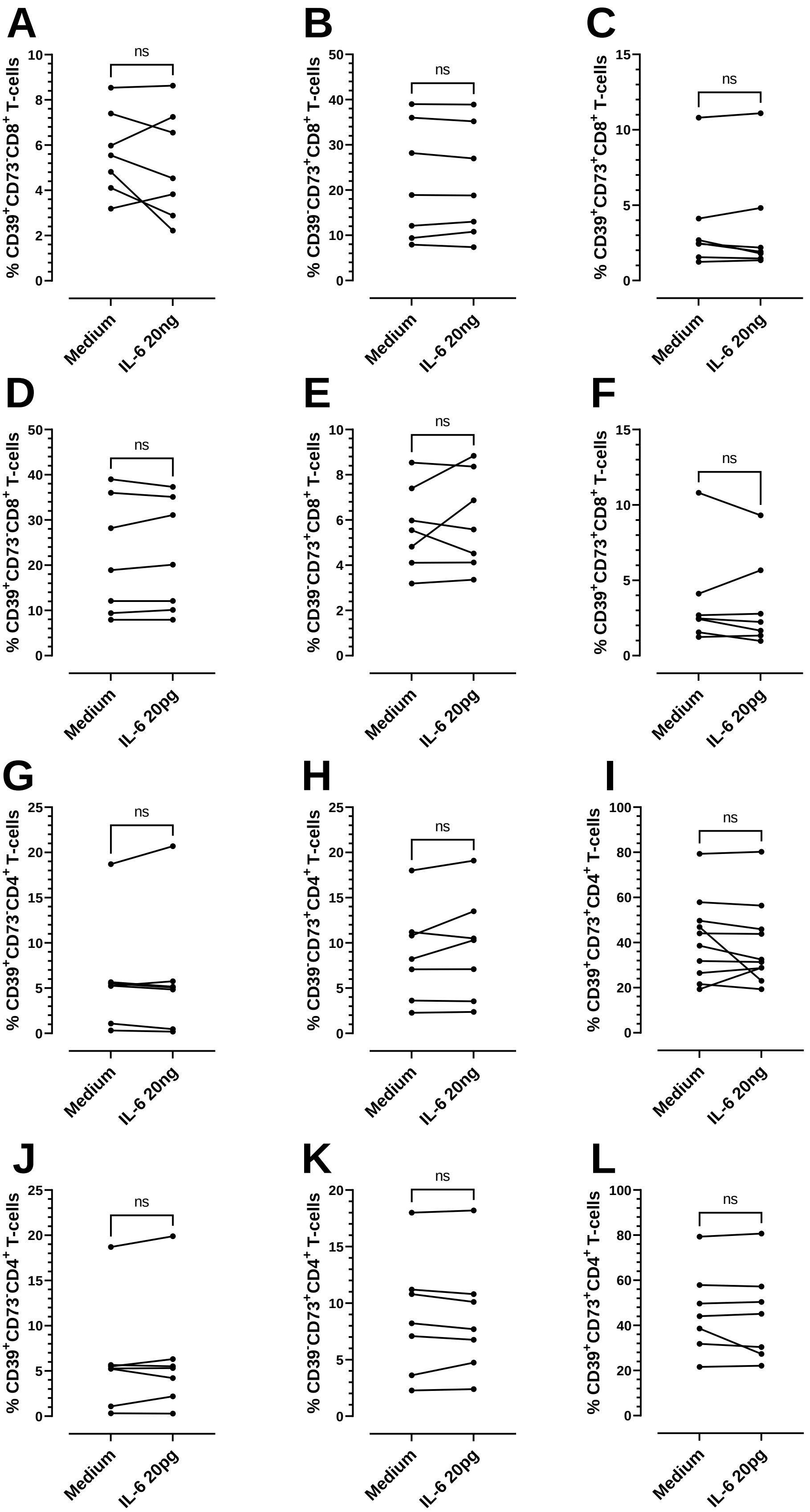

Supplementary Figure 2. Frequency of CD39+ or CD73+ cells gated in CD8+ T-cells (A-F) or gated in CD4+ T-cells (G-L) stimulated with recombinant IL-6 (20 ng/mL or 20 pg/mL) or maintained in medium. ns: not significant.
